# Create your own stimulus: Manipulating movements according to social categories

**DOI:** 10.1371/journal.pone.0174422

**Published:** 2017-03-24

**Authors:** Markus Koppensteiner, Georg Primes, Pia Stephan

**Affiliations:** 1 Department of Anthropology, University of Vienna, Vienna, Austria; 2 Netherlands Institute for Advanced Study (NIAS), Royal Netherlands Academy of Arts and Sciences (KNAW), Amsterdam, The Netherlands; University of Münster, GERMANY

## Abstract

People ascribe purposeful behaviour to the movements of artificial objects and social qualities to human body motion. We investigated how people associate simple motion cues with social categories. For a first rating-experiment we converted the body movements of speakers into stick-figure animations; for a second rating-experiment we used animations of one single dot. Rating-experiments were “reversed” because we asked participants to alter the movements (i.e., vertical amplitude, horizontal amplitude, and velocity) of the stimuli according to different instructions (e.g., create a stimulus of high dominance). Participants equipped stick figures and dot animations with expansive movements to represent high dominance. Expansive and fast movements (i.e., high velocity) were mainly associated with high aggressiveness. Fast movements were also associated with low friendliness, low trustworthiness, and low competence. Overall, patterns found for stick figure and dot animations were similar indicating that certain motion cues convey social information even when only a dot and no body form is visible. The “reverse approach” we propose here makes the impact of different components directly observable. The data generated by this method offers better insights into the interplay of these components and the ways in which they form meaningful patterns. The proposed method can be extended to other types of nonverbal cues and a variety of social categories.

## Introduction

Motion means change. Changes in the physical and social environment can have positive or negative consequences for an organism. For this reason, quickly detecting and categorizing motion and motion cues can be regarded as an important ability that helps to assess a situation and to make predictions. Indeed, people’s propensity to perceive motion as a mediator of relevant information is strong and makes them attribute personality traits to featureless moving objects or interpret the movements of these objects as purposeful and as being of animal or human origin [1–4]. For instance, two artificial arrow-headed objects interacting on a screen can be understood as living beings chasing each other, courting, or fighting [5]. In conclusion, although people tend to over-generalize in interpreting motion as purposeful behaviour, such misinterpretations may be less costly than ignoring potentially relevant information.

As a social species, humans pay close attention to their conspecifics and try to make sense of non-verbal cues conveyed by different communication channels [6]. Considering this, and the propensity to attach intentions and meaning to simple moving dots, it is not surprising that people are sensitive to the movements created by a human body. In a now classical experiment, Johannsson filmed people wearing reflective markers in front of a dark background and found that the resulting videos showing only moving dots preserved enough information for the recognition of a human body [7]. Such so called “point light” displays have also been successfully used to demonstrate that people can make quite accurate guesses about a point-light-walkers’ gender and age [8,9].

Applying the “point -light” technique and other methods of capturing or describing movements has revealed that motion patterns created by a wide range of human behaviours such as walking, dancing, and gesturing are carriers of social information. For instance, human gait and simple arm movements appear to contain enough variation to inform about emotional states and personality [10–12]. People are able to recognize emotions in body movements that are displayed by actors [13–16] and ascribe different intentions and interpersonal qualities to actors’ displays of hero or villain like body movements [17]. Also, emotions and personality traits are expressed through motion patterns shown during dance performances [18–20]. Body motion affects the impressions people form about potential mating partners [21], politicians giving a speech [22–24] and public speakers in general [25].

In the current study, we focused on the communicative value of body motion. We investigated how people associate a set of specific motion cues with a selection of items representing basic interpersonal categories. In contrast to the “classical” approach of performing rating-experiments we turned the rating procedure upside down. Instead of asking participants to judge unmodifiable stimuli on different scales, we asked them to modify the appearance of the stimuli they saw according to a set of verbal descriptors. In other words, people took a more active role in our experiments because they were directly involved in stimulus creation. In our view such an approach has several advantages. First, it allows a greater control over testing how specific cues affect social judgements because only these specific cues are modified during the experiments. Second, these modifications are done by the participants themselves and not by an experimenter. Third, the approach makes it easier to examine how single cues combine to generate meaningful patterns.

To create the stimuli for the current study we relied on material we already had collected in previous studies [22,26,27]. For these studies we extracted short excerpts from videos showing speakers of the German Parliament, we encoded the movements of specific body parts (e.g., hands, head) and we used the resulting coordinate data to translate the body movements into stick-figure animations. Thus, the source material for the stimuli used here was based on movements that are free from experimental interventions (i.e., no actors involved) and similar to the point-light displays—free from confounding variables such as appearance cues. In a second step, we only extracted the movements of the right hand of the speakers and reduced them to one single moving dot. This was done to test whether the social categories we used can be related to motion patterns that lack information about body form.

Subsets of the stimuli served as raw material for a “reverse” rating experiment. This means that we did not ask participants to judge a set of given stimuli on different items. Instead, we presented an item (i.e., randomly selected from a set of items) to our participants and asked them to match the motion behaviours of the stimuli (i.e., stimuli were composed of different stick-figures) with the presented item. To perform their modifications, the participants used different slider bars (i.e., graphical control elements on a computer interface to set values by moving an indicator), each changing one aspect of a stimulus’ motion behaviour. Consequently, within certain boundaries the “reverse” approach enabled the participants to create”their “own stimuli and enabled us to examine whether specific elements of body motion (i.e., vertical and horizontal movements and their velocity) can be isolated as carriers of interpersonal information.

Perceiving and inferring personality traits and different social qualities in other people’s appearance or behaviours may be an ability that has developed through the course of human evolution. It helped to assess, in a general way, the intentions of a potential interaction partner and to make quick decisions regarding whether to approach or avoid somebody [6,28–30]. Given that people form such impressions so quickly, it is clear that these are rough estimates on the basis of broad social categories. Research using facial photographs has found that people relate likability, competence, trustworthiness, dominance, and aggressiveness to facial appearance [30,31]. We modelled the items of our experiment after such work and we asked the participants to adapt body movements to positive and negative expressions of the adjectives friendly, dominant, aggressive, trustworthy, and competent (e.g., competent and not competent).

Power and dominance appear to be conveyed through expansive body postures, expressive body movements, and broad gestures [23,32–36]. Hence, we expected high dominance and high aggressiveness to be associated with more expansive movements and higher velocity than low dominance and low aggressiveness. High friendliness and high trustworthiness were assumed to be associated with less expansive and slower movements than low friendliness and low trustworthiness because in previous research using stick-figure animations of speakers we found that both social categories are negatively related with dominance [22]. Using the methodical framework of classic rating studies, in this previous work we also found no clear relationship between competence and the body motion of speakers [23]. Therefore, we were unable to formulate a hypothesis on how this social category is reflected in the motion components that were manipulated here.

To sum up, we investigated the role of simple motion cues in social categorizations. We redesigned the common procedure of performing rating-experiments by giving the participants of our experiments the opportunity to directly manipulate the appearance of a stimulus. The participants were asked to change the movements of stick-figure animations and single dot animations in accordance with a given set of adjectives belonging to different social categories. This experimental set-up allowed us to determine specifically the role of certain motion cues (i.e., horizontal and vertical expansiveness as well as velocity) in impression formation. Stick-figure animations preserve some information about body form and about the movements of certain body parts. However, there is evidence that motion patterns are carriers of social information even when it is not apparent that they are of human or animal origin. Therefore, we also used single dot animations in order to determine whether information about the social classifiers we used can be conveyed independently of body form.

## Material and methods

### Ethics statement

The Experiments were conducted in accordance with the Declaration of Helsinki (revised 1983) as well as local guidelines of the Faculty of Life Sciences, University of Vienna. In accordance with the Austrian Universities Act 2002 (UG2002), in place at the time the study was carried out, only medical universities were required to appoint ethics committees for clinical tests, application of medical methods, and applied medical research. As a result, no ethical approval was required for the present study. We did not collect personal data except age and gender. Experimental data, which were identified by numeric codes, were not personally traceable and therefore a written consent by the participants was not required.

### Participants

Data collection was part of a practical course in human behaviour research taking place at the University of Vienna’s Department of Anthropology (in November 2015). Students of this course asked people at locations throughout the university campus to take part in our experiments. 20 females and 22 males (age *M* = 22.79 years, *SD* = 3.24) participated in the first experiment during which we used stick-figure animations as stimuli. For the second experiment, during which people were asked to modify the appearance of single dot animations, a different sample as in the first experiment was recruited. Twenty different females and 20 males (age *M* = 22.43 years, *SD* = 5.07) participated in this second experiment. People participated voluntarily in the experiments and were not reimbursed.

### Stimulus preparation

#### Source material

**Fig 1:**
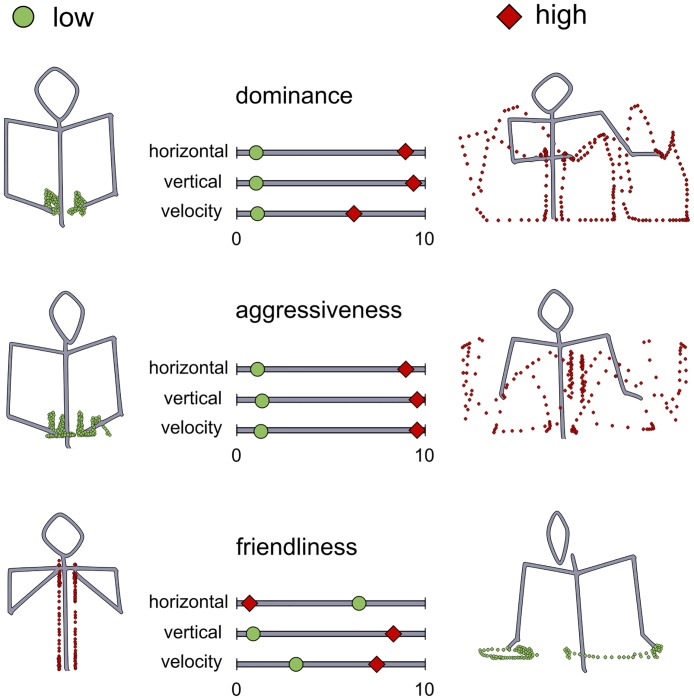
Examples for the motion manipulations participants performed during the experiments. On the left “low” versions of social categories are presented. On the right “high” versions are presented. Slider bar positions that corresponded with manipulation of motion are shown in the centre. Points give an impression of the arm-movements displayed.

**Fig 2:**
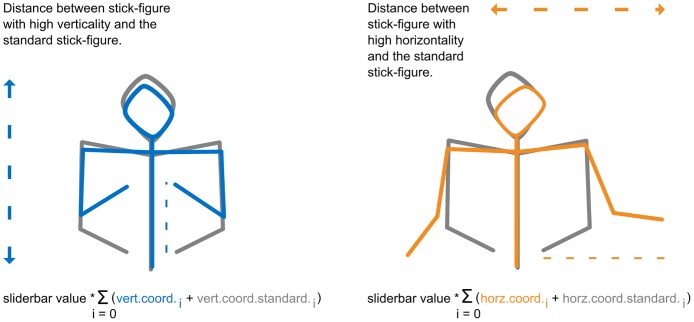
For the experiment stick-figures showing pronounced vertical movements (but no horizontal movements) were combined with stick-figures showing pronounced horizontal movements (no vertical movements). To enable manipulation of the vertical amplitudes the software calculated a weighted average (weight was equal to slider bar value modified by participants) between the y-coordinates of the standard stick figure (vert.coord.standard) and the stick-figures of high verticality (vert.coord.). Manipulation of the horizontal amplitudes followed the same principle. A weighted average between x-coordinates of stick-figures showing high horizontality (horz.coord.) and the standard stick-figure (horz.coord.standard) was calculated. Calculations were done for all of the twelve landmark-coordinates (index i) and all frames.

Stimuli for both experiments (i.e., stick-figure and single dot animations) were based on brief, randomly selected video sequences (15 s) taken from 100 speeches (50 male and 50 female speakers) that had been collected for previous studies. All sequences had the same video resolution (i.e., 768 x 432 pixels) and the same frame-rate (i.e., 25 frames per second). The speakers were members of the German Parliament whose body movements were tracked by rearranging landmarks (i.e., dots that were moved by drag and drop operations) according to the position shifts of specific body parts (e.g., head, hands, throat etc.). All such position shifts during a video yielded a set of time series of two-dimensional coordinates. These time series were used to create animated stick-figures (Figs 1 and 2) that served as abstract representations of the speaker’s body movements (for more information on these procedures see [22,26,27]).

All speakers in the videos were shown in a frontal view and at nearly the same distances from the camera. Nevertheless, it was necessary to mitigate confounding influences due to differences in body heights and limb lengths. To accomplish this we first measured the height of each stick-figure by determining the maximal vertical distance between the forehead landmark and the centre of gravity landmark. Then we defined a standard height (i.e., 275 pixels) and recalculated the landmark coordinates of all stick-figures on the basis of this standard height (the details of this procedure are presented in the folder stimulus preparation of S2 Dataset). In short, all stick-figure coordinates of all frames were resized, so that the maximal stretch (i.e., maximal distance between forehead and centre gravity) was the same for each stick-figure. To position all stick-figures at nearly the same spot on the screen we shifted them to a standard position. More precisely, at the maximum stretch the centre of gravity of each stick-figure was 345 pixel (horizontal direction) and 341 (vertical direction) from the coordinate origin.

#### Selection of subsets from the source material

From the resized (see above) coordinate data of each of the 100 encoded speeches we chose sequences (5 s) with pronounced expansive vertical and expansive horizontal movements. From these we selected the ten sequences showing the most pronounced vertical movements and the ten sequences showing the most pronounced horizontal movements. Single steps of these procedures are explained below.

First, we determined the vertical distance (using only y-coordinates) between the right hand and the desk, the left hand and the desk, and the distance between the throat and the desk for each frame of the available coordinate data. Motion information contained in these distances (i.e., amplitudes) was summed for each encoded frame yielding a simple estimator for the overall vertical expansiveness.

Second, we summed amplitudes obtained by calculating the horizontal distances (using only x-coordinates) between the right hand and the throat, the left hand and the throat, and the distance between the throat and the coordinate origin. This yielded an estimator for the horizontal expansiveness of each encoded frame.

Third, for each speech we determined the sequence with a length of five seconds for which vertical or horizontal expansiveness was maximal. This resulted in 200 five seconds long sequences (i.e., 100 for horizontal motion and 100 for vertical motion). These sequences were ranked from maximal to minimal horizontal and vertical expansiveness. We then chose coordinate data of ten stick-figures with very expansive vertical movements and coordinate data of ten stick-figures with very expansive horizontal movements as a subset for prototype creation.

In the following we present the range of motion for the ten high rankers on overall verticality and the range of motion for the ten high rankers on overall horizontality (i.e., measures that were used for the selection process), as well as the range of the elements both measures are composed of.

The maximal overall vertical amplitudes ranged from 271 to 405 pixels (see procedure for information about resolution of screen). The maximal vertical amplitudes of the anatomically right hand ranged from 137 to 204 pixels, those of the left hand ranged from 193 to 204 pixels and the maximal vertical amplitude of the upper body ranged from 6 to 39 pixels.

The maximal overall horizontal amplitude ranged from 261 to 469 pixels. The maximal amplitudes of the right hand ranged from 92 to 243 pixels, those of the left hand ranged from 117 to 295 pixels and the maximal horizontal amplitudes of the upper body ranged from 15 to 166 pixels.

For our second experiment, during which we reduced animations to one single moving dot, we only used the amplitudes of the anatomically right hand. For prototype creation we again extracted ten sequences of very expansive vertical and ten sequences of very expansive horizontal movements. We chose the right hand because we assumed that for most speakers the right hand was the dominant one and therefore was used preferably. The maximal vertical amplitude of the right hand for the subset of stimuli used for the dot-animations ranged from 138 to 196 pixels and the maximal amplitude of horizontal motion ranged from 159 to 275 pixels.

We also made sure that the subsets of high vertical and high horizontal expansiveness contained different stick-figures in each experimental condition. However, there was some overlap between the conditions. Five stimuli with high vertical expansiveness and two with high horizontal expansiveness were not only used in the stick-figure animation experiment but also in the dot-animation experiment.

#### Creating prototypes for vertical and horizontal motion

The selected subsets of ten stick-figures and ten dot-animations were turned into animations showing only vertical movements and animations showing only horizontal movements.

To create stick-figures showing only vertical but no horizontal movements we replaced the x-coordinates of all frames with the x-coordinates of a “standard” stick-figure. The “standard” stick-figure was positioned in the middle of the desk with both hands held close together at the height of the navel. After this, x-coordinates from the first to the final frame had the same values, while y-coordinates still showed variation. Similarly, to create stick-figures showing only horizontal motion and no vertical motion we replaced the y-coordinates of all frames with the y-coordinates of the “standard” stick-figure (see Fig 2, S1 and S2 Videos).

#### Manipulation of horizontal and vertical amplitudes

Manipulated coordinate data of both categories (i.e., “prototypes” of vertical and horizontal movements only) served as raw material for our “reverse” rating experiments. For each round of an experimental session, a randomly chosen data-set representing vertical expansiveness was merged with a randomly chosen data-set representing horizontal expansiveness. Overall, this gave 100 possible combinations (ten vertical by ten horizontal high rankers on motion).

For the manipulation of the stimuli’s vertical amplitude, horizontal amplitude, and velocity, three slider bars were positioned on the right hand side of the rating program. Slider bars are a common element in program interfaces that allow changing a numerical value by moving a bar between two poles using the computer-mouse.

On the basis of the slider bar values, the program calculated a weighted mean between the coordinates of the “prototypes” and the coordinates of the standard stick-figure (Fig 2, S1 Video, software and data in S2 Dataset). For instance, moving the slider bar named “vertical amplitude” to the left pole (i.e., equals zero) removed all vertical movements (i.e., sets y-coordinates of each frame to coordinates of “standard” stick-figure), while moving the slider bar to the right pole showed the maximum amplitude of vertical movements (i.e., sets y-coordinate of each frame to original coordinates of stick-figure showing expansive vertical movements). Thus, motion manipulation during the experiment ranged from a total damping of movements to no damping of movements; in between coordinates were a weighted mean with the slider bar values (ranging from zero to one in steps of 0.1) being the weight (Fig 2 and software in S2 dataset).

The velocity of body motion was changed by raising or lowering the frame rate of the animation. Moving the bar to the far left of this slider control changed the frame rate to 25 frames per second while moving it to the far right raised the frame rate to 58 frames per second (in steps of three frames per second), thereby making overall motion of the stick-figures appear faster (Fig 2 and software in S2 Dataset).

Although our raw data was based on “natural” behaviours, we, of course, added an element of artificiality by combining behaviours from different sources (i.e., mixing data from two stick-figures) and raising the frame-rate of the animations. It has already been shown that linear interpolation of two movement patterns often leads to “naturally” looking outcomes with small contortions only and no impossible movements [37]. Hence, we also relied on such a method of interpolation. This maintained a “natural flow” by preserving central properties of body motion (e.g., hand movements in harmony with movements of the rest of the body and vice versa, movements that are anatomically correct etc.) which are hard to reproduce artificially, and also kept manipulations within reasonable boundaries.

### Procedure

For both rating experiments, students of a practical course in human ethology approached potential participants at the University and invited them to take part in a short experiment. The participants were brought into a separate room with several computers. They were seated in front of one of the computers and received instructions on how to use the rating program. The stimuli were displayed on 15.6 inch screens at a resolution of 1366 x 768 pixels. The interface of the rating program contained an animation window, which was located in the upper left corner. A button named “START” was below the animation window and to the right of it were three slider bars (see demo-program in S2 Dataset). The size of the animation window was 768 x 432 pixels. In both experiments (i.e., stick-figure animations and dot animations), people were asked to change the movements of the presented stimuli according to five different items. The items were named after the German versions of dominant, trustworthy, friendly, aggressive and competent and were presented in a “low” and a “high” version (e.g., low dominance versus high dominance). The items were displayed above the three slider control bars and embedded in the following instruction (translated into English): “Please, adjust the movements of the stimulus so it appears to be very dominant”. Slider bar values ranged from zero to ten (converted to values between zero and one for interpolation) enabling participants to adjust vertical amplitudes, horizontal amplitudes, and velocity on eleven different levels. Each round of an experimental session started with a composition of a randomly selected stick-figure with high horizontal expansiveness and a randomly selected stick-figure with high vertical expansiveness. The values of all sliders were set to the central position at the beginning of each round. Consequently, the stimulus at the beginning was an average of the two selected stick-figures (see S2 Video). Moving a slider bar had an immediate effect on the appearance of the stimulus (i.e., all coordinates of all frames were recalculated). After having made their adjustments the participants clicked on the button “NEXT” to move on to the next stimulus. The button “NEXT” was unlocked only after the participants had moved all three slider bars, so that they could not proceed without having made any changes. Each experimental session consisted of ten rounds: one round for each version of the five items. The items were presented in random order. The participants had no time limit; they switched from one stimulus to the next by clicking on the button in the main window. A demo version of the rating program can be found in S2 Dataset.

### Analyses

Slider bar values were used as predictors in a logistic regression with “high” and “low” versions of the items as binomial outcome variable (e.g., very dominant encoded as 1 and not dominant as 0). Interpretation of the data focused strongly on the estimates of the regression analyses, as they give insight into the relative contribution of each independent variable.

When predictors are strongly correlated they might be affected by multicollinearity. Multicollinearity can distort the regression coefficients and make them uninterpretable. Thus, to guard against such misinterpretations we also present regression models on the basis of one predictor only (e.g., slider bar values of horizontal movements) and so-called relative weights. Relative weights or relative importance weights range between 0 and 1, are unaffected by multicollinearity, and therefore are an efficient effect size measure that helps interpreting the estimates of a regression analysis [38,39]. They give insight in the proportionate contribution from each predictor to overall variance of a model by correcting for the effects of inter-correlations among predictors. This method is highly valuable when it is important to know the relative contribution each predictor variable makes to the dependent variable. The proportion given by a relative weight corresponds with the explained variance (*r*^2^) of a specific predictor. Adding up the relative weights of a subset of all predictors provides the explained variance of this subset (e.g., explained variance of vertical motion and horizontal motion together) and adding up all relative weights provides the overall explained variance (*R*^2^) of a regression model. Moreover, we also present inter-correlations between the predictors of those regression models, where multicollinearity is suspected (i.e., where regression estimates and relative weights lead to contradictory interpretations).

We also present 95% confidence intervals for the regression estimates. By inspecting the overlaps of the confidence intervals it is possible to determine whether there are noteworthy differences between the estimates of different regression models. In interpreting these overlaps we follow the guidelines of Cumming and Finch [40]. For instance, when confidence intervals only touch or show no overlap this is equal to a *p*-value below .01.

All statistical analyses were carried out in the program R [41]. R-code (also with the code for calculating relative weights) can be found in S1 Dataset.

## Results

Some of the results we obtained are shown in Fig 1. The examples presented in this figure are based on motion manipulations performed by single participants and therefore only serve illustration purposes.

### Descriptive statistics

Descriptive statistics for the first experiment, during which we used stick-figure animations, are shown in Table 1. Descriptive statistics for the second experiment, during which we used single dot animations, are shown in Table 2. Because the data deviated from the normal distribution, we used medians as measures of central tendency. Hence, both tables contain people’s median slider bar manipulations (i.e., manipulations of horizontal amplitudes, vertical amplitudes and velocity) for each of the different verbal categories (e.g., high dominance). For some social categories the motion components showed a higher variation (i.e., greater range spanned by first and third quantile) than for other social categories. Therefore, it seems that the participants experienced difficulty to make relatively clear decisions on which motion patterns to choose for some of the verbal descriptors. For instance, agreement among the participants in adjusting the vertical amplitude for low stick-figure dominance was stronger than the agreement in adjusting the vertical amplitude for low trustworthiness (Table 1).

**Table 1:** Descriptive statistics for stick-figure animation experiment.

Categories	Motion Components		
	Horizontal Amplitude	Vertical Amplitude	Velocity
High Dominance	7 (4;8)	7 (5.25;10)	6 (4;8)
Low Dominance	1 (0;3.75)	1 (1;3)	2.5 (1;5.75)
High Aggressiveness	9 (6;10)	10 (9;10)	10 (8;10)
Low Aggressiveness	2.5 (1;5)	2 (1.25;6)	2 (2;4)
High Trustworthiness	4 (2;6)	4 (3;6)	3 (2;4)
Low Trustworthiness	7 (2;9)	6 (2;9)	7 (4;9)
High Competence	4.5 (3;6)	5 (3;6)	4 (3;5)
Low Competence	6 (3;10)	4 (1;7)	7 (3;9.75)
High Friendliness	4 (3;6)	4 (3;6)	3 (3;7)
Low Friendliness	6 (3;9)	8 (5;9)	7.5 (5.25;9.75)

*Note*. Median slider bar values with first and third quartile in parenthesis. Slider bar values ranged from 0 to 10 with 0 meaning low amplitude or velocity and 10 meaning high amplitude or velocity. *N* = 42.

**Table 2:** Descriptive statistics for dot animation experiment.

Categories	Motion Components		
	Horizontal Amplitude	Vertical Amplitude	Velocity
High Dominance	5 (2.75;9)	8 (7;10)	6 (3;8)
Low Dominance	2 (1.75;3)	1.5 (1;2)	2 (1;3)
High Aggressiveness	8.5 (3.75;10)	9 (7;10)	10 (10;10)
Low Aggressiveness	3 (2;4.25)	1 (1;3)	1 (0;2.25)
High Trustworthiness	5 (2;7)	2.5 (1.75;4)	2 (1;3)
Low Trustworthiness	7.5 (4.5;9)	6 (2;8)	7 (3;9.25)
High Competence	5 (3;7)	5 (2;7)	3 (2;5)
Low Competence	6 (4;9)	5 (1;8)	7 (2;9)
High Friendliness	5 (2.75;6.25)	5 (3;7)	3 (2;5)
Low Friendliness	6.5 (4;9)	5 (2;7)	7.5 (6;9)

*Note*. Median slider bar values with first and third quartile in parenthesis. Slider bar values ranged from 0 to 10 with 0 meaning low amplitude or velocity and 10 meaning high amplitude or velocity. *N* = 40.

### Logistic regressions for stick-figure animations

People had been asked to adjust the horizontal amplitude, the vertical amplitude, and the velocity of a stick figure animation to positive and negative expressions of different social categories (e.g., high dominance versus low dominance). We performed logistic regression analyses using high and low dominance as binary outcome variables and the motion components as predictors (Table 3). Our interpretations of the data rely on the relative contributions of these predictors. However, interdependencies between predictors of multiple regressions can lead to multicollinearity and as a consequence to misleading results. For this reason, our conclusions are mainly based on relative weights (see Method/Analyses), which are unaffected by multicollinearity. In addition to this we present the regression estimates of logistic regressions that are based on single predictors (e.g., only horizontal movement) only (Table 4).

**Table 3:** Logistic regressions of experiment with stick-figure animations.

Social Categories	Motion Components			
	Horizontal Amplitude	Vertical Amplitude	Velocity	*R*^*2*^
Dominance	.21 (.11)	.43 (.12)	.07 (.12)	.54
	[-.01, .43]	[.22, .69]	[-.17, .30]	
	**.16**	**.29**	**.09**	
Aggressiveness	.29 (.13)	.30 (.14)	.58 (.16)	.75
	[.04, .57]	[.01, .60]	[.31, .95]	
	**.18**	**.24**	**.33**	
Trustworthiness	-.16 (.10)	-.05 (.10)	-.47* (.12)	.37
	[-.36, .03]	[-.25, .16]	[-.73, -.26]	
	**.07**	**.02**	**.28**	
Competence	-.14 (.08)	.06 (.09)	-.17 (.08)	.10
	[-.30, .02]	[-.11, .24]	[-.33, -.02]	
	**.04**	**.00**	**.06**	
Friendliness	-.08 (.09)	-.21 (.09)	-.26 (.10)	.25
	[-.26, .10]	[-.39, -.03]	[-.46, -.08]	
	**.02**	**.10**	**.13**	

*Note*. Regression estimates and standard errors (in parenthesis); 95% confidence intervals in brackets. Relatives Weights giving explained variance unaffected by other predictors in bold. *N* = 42;

* *p* < .0017;

*p*-values were Bonferroni corrected (30 tests).

**Table 4:** Logistic regressions for stick-figure animations using motion parameters as single predictors.

Social Categories	Motion Components		
	Horizontal Amplitude	Vertical Amplitude	Velocity
Dominance	.41* (.09)	.56* (.11)	.33* (.09)
	[.25, .60]	[.37, .81]	[.17, .51]
Aggressiveness	.38* (.08)	.55* (.11)	.78* (.15)
	[.23, .56]	[.36, .79]	[.52, 1.12]
Trustworthiness	-.21 (.08)	-.12 (.08)	-.51* (.12)
	[-.36, -.06]	[-.28, .03]	[-.76, -.30]
Competence	-.14 (.07)	.02 (.08)	-.18 (.08)
	[-.29, .00]	[-.13, .18]	[-.34, -.03]
Friendliness	-.14 (.08)	-.28 (.09)	-.34 (.09)
	[-.30, .00]	[-.46, -.12]	[-.53, -.17]

*Note*. Motion parameters were used as single predictors in regression analyses in order to test their impact on the criterion without considering other variables. Table shows regression estimates and standard errors (in parenthesis) and 95% confidence intervals (in brackets). *N* = 42;

* *p* < .0017;

*p*-values were Bonferroni corrected (30 tests).

An inspection of the relative weights for dominance revealed that vertical motion explained most of the overall variance, followed by horizontal motion, and that velocity is in last position (Table 3). Regression estimates (i.e., beta weights) replicated this pattern, but by the standards of null hypothesis significance testing none of the variables yielded a noteworthy result. This changed when we created three independent regression models for dominance, each of which with a different motion component as single predictor (Table 4). It appears that in the multiple predictor regression model for dominance (Table 3) interdependencies between the predictors (i.e., multicollinearity) affected the results. Indeed, the correlation between horizontal and vertical motion was *r* = 58, that between vertical motion and velocity was *r* = 57 and that between horizontal motion and velocity was *r* = .51. Consequently, a highly dominant stick-figure tended to have high values on all three motion parameters. This is also reflected in the relative weights and the size of the beta-weights obtained from single predictor regressions. In short, our participants tended to equip high dominance stick-figures with faster and more expansive vertical and horizontal movements than low dominance stick-figures.

A similar result as for dominance was obtained for high and low aggressiveness. However, in this case, velocity was the most important predictor. More precisely, participants tended to equip animations they were asked to turn into highly aggressive stick-figures with expansive vertical and horizontal movements and a high velocity of motion. Taking a closer look at the regression estimates of the single predictor models reveals that the confidence intervals for the velocity “ratings” of dominance and aggressiveness do not overlap (i.e., would result in a *p*-level below .01), while those for horizontal and vertical motion nearly cover the same range (Table 4). This underlines that velocity played a more prominent role for animations that were turned into stick-figures displaying low and high aggressiveness than for animations that were turned into stick-figures displaying high and low dominance.

Overall, velocity was a predominant influential factor. Inspection of multiple and single predictor regression models (fourth column in Tables 3 and 4) shows that the sign of the estimates for velocity is positive for dominance and aggressiveness, while it is negative for all other social categories. These findings demonstrate that velocity of motion had a wide-ranging but no uniform impact on the decisions people made during the experiment. Faster movements were preferably associated with high aggressiveness and high dominance, but also, on the other hand, with low friendliness, low trustworthiness, and low competence.

Besides velocity expansive vertical movements played an additional role for friendliness. Therefore, expansive and fast vertical movements did not go together with high friendliness. Moreover, there was a tendency to associate high trustworthiness with less expansive horizontal movements. However, the effect size for this is not very pronounced.

Aggressiveness and friendliness are perceived as mutually exclusive categories, yet they did not show mutually exclusive motion patterns. Horizontal movements played no role in “ratings” of friendliness but in “ratings” of aggressiveness. In addition, high friendliness and low friendliness created less pronounced differences and smaller effect sizes for vertical motion and velocity than low and high aggressiveness. Thus, both motion components were more important for aggressiveness than for friendliness; nevertheless, these two social categories were intertwined.

Regression models for trustworthiness, friendliness and competence explained less overall variance than those for dominance and aggressiveness. This hints that variations in the motion components that were manipulated during the experiments are mainly conveying information about aggressiveness and dominance. Regression models for competence explained the smallest proportion of variance. In comparison to the other social categories it was apparently more difficult for the participants to create a stimulus that conveys competence.

### Logistic regressions for dot animations

In a second experiment people modified the movements of a simple dot-animation. As in the first experiment interpretations of the results are mainly based on the relative weights of the multiple predictor regressions (Table 5) and the regression estimates of the single predictor regression models (Table 6). Findings of the dot animation experiment resembled those of the stick-figure animation experiment. However, the results for the dot animations showed more variation (Tables 1 and 2) than those for the stick-figure animations. The absence of body form information apparently makes the task for the participants more difficult.

**Table 5:** Logistic regressions of experiment with dot animations.

Social Categories	Estimates			
	Horizontal Amplitude	Vertical Amplitude	Velocity	*R*^*2*^
Dominance	.31 (.13)	.60* (.13)	.26 (.13)	.65
	[.07, .59]	[.37, .91]	[.02, .54]	
	**.14**	**.39**	**.12**	
Aggressiveness	.30 (.26)	.50 (.34)	.76 (.23)	.93
	[-.18, .92]	[-.06, 1.43]	[.41, 1.36]	
	**.15**	**.35**	**.43**	
Trustworthiness	-.12 (.11)	-.14(.12)	-.51* (.12)	.43
	[-.35, .09]	[-.38, .09]	[-.78, -.29]	
	**.05**	**.08**	**.30**	
Competence	.06 (.09)	.09 (.08)	-.33 (.10)	.18
	[-.12, .25]	[-.07, .27]	[-.55, -.15]	
	**.01**	**.01**	**.16**	
Friendliness	-.03 (.11)	.08 (.10)	-.59* (.14)	.38
	[-.24, .19]	[-.12, .30]	[-.90, -.35]	
	**.03**	**.00**	**.35**	

*Note*. Regression estimates and standard errors (in parenthesis); 95% confidence intervals in brackets; Relatives Weights giving explained variance unaffected by other predictors in bold. *N* = 40;

* *p* < .0017;

*p*-values were Bonferroni corrected (30 tests).

**Table 6:** Logistic regressions for dot animations using motion parameters as single predictors.

Social Categories	Motion Components		
	Horizontal Amplitude	Vertical Amplitude	Velocity
Dominance	.32 (.09)	.61* (.12)	.31 (.09)
	[.15, .51]	[.41, .87]	[.15, .50]
Aggressiveness	.35* (.08)	.70* (.14)	1.00* (.22)
	[.20, .53]	[.47, 1.02]	[.64, 1.58]
Trustworthiness	-.20 (.08)	-.28 (.09)	-.53* (.11)
	[-.36, -.05]	[-.47, -.12]	[-.79, -.33]
Competence	-.08 (.07)	.01 (.07)	-.27 (.08)
	[-.23, .06]	[-.13, .14]	[-.44, -.12]
Friendliness	-.17 (.08)	-.03 (.08)	-.58* (.13)
	[-.34, -.01]	[-.19, .12]	[-.86, -.35]

*Note*. Motion parameters were used as single predictors in regression analyses in order to test their impact on the criterion without considering other variables. Table shows regression estimates, standard errors (in parenthesis) and 95% confidence intervals (in brackets). *N* = 40;

* *p* < .0017;

*p*-values were Bonferroni corrected (30 tests).

As in the stick-figure condition dominance and aggressiveness yielded the most convincing results. Again expansiveness of vertical movements was a prominent predictor for animations intended to represent dominance or aggressiveness. Horizontal expansiveness played a minor but still substantial role. The raw values of high dominance horizontal motion show more variation than the raw values of low dominance horizontal motion (Table 2). It appears that it was difficult for the participants to relate dot-animations of high dominance to horizontal motion. To a certain extent this undermines the predictive power of horizontal motion. The largest difference between dominance and aggressiveness was again found for velocity. Participants of our experiment associated fast movements more strongly with high aggressiveness than high dominance (see relative weights of Table 5 and regression estimates and non-overlapping confidence intervals in Table 6).

Multicollinearity appeared to affect results for aggressiveness. In depth analyses of interdependencies between the predictors revealed a correlation of *r* = .34 between horizontal and vertical motion, a correlation of *r* = .71 between vertical motion and velocity and a correlation of *r* = .45 between horizontal motion and velocity. Thus, for ratings of aggressiveness there was a strong link between vertical motion and velocity. In other words, fast as well as expansive up and down movements appeared to produce impressions of high aggressiveness. Overall, as in the first experiment expansiveness of motion and its velocity were strongly related to dominance and aggressiveness. Because we only used a single dot as stimulus in the second experiment, such findings indicate that some cues creating impressions of dominance and aggressiveness are independent of body form.

For the rest of the verbal categories, velocity was revealed as meaningful predictor. As in the first experiment fast movements were associated with low friendliness, low competence, and low trustworthiness. However, there were also some differences. Vertical movements, that were important to differentiate between friendly and unfriendly stick-figure animations, played no role in differentiating between friendly and unfriendly dot animations. It is possible that people need to see arms to associate low friendliness with expansive vertical motion. Unlike in the first experiment low and high trustworthiness yielded a noteworthy effect size for vertical amplitude. These conflicting findings require additional studies, in which the interaction between body form and motion cues needs to be examined in greater depth. For instance, removal of form information could be done in a stepwise manner by using different levels of abstraction (e.g., full body, arm only, dot only).

## Discussion

People are well-versed in forming social information from the movements of a body. The propensity to attribute meaning to motion is so strong that the movements of artificial objects are often perceived as human or animal behaviour. In the current study, we elaborated on this and we pursued two aims. First, we tested the applicability of a “reverse” rating experiment, during which participants built their own stimuli by changing movements until the stimuli appeared to be accordance with a given item. Second, we compared manipulations of stick-figure animations, which still give an impression of body form, with manipulations of single dot animations, in order to test whether people make similar adjustments even when information about body form is missing.

The ability to judge other people quickly by nonverbal cues may be due to evolutionary pressures, as it has been beneficial to decide quickly between “friend” or “foe” or to form expectations about one’s social environment [42,43]. On the signal sender’s part, some emotional states and social intentions are communicated relatively unambiguously and are visible in overt behaviours. For instance, dominance displays are not subtle and are expressed through expansive body postures and body movements [36]. For this reason, we expected our participants to equip stimuli that should appear dominant with expansive movements. Indeed, our findings were in accordance with these expectations. Participants associated expansive horizontal and vertical movements with high dominance for both stick-figures and dot-animations. Such findings not only support previous work but also show that some dominance cues are embedded in the behavioural stream and do not lose their impact even when the body that produces them is not fully visible.

A similar reasoning as above applies to aggressiveness. Those who detect aggression quickly may be one step ahead avoiding or countering aggressors, while the aggressors may assert themselves by turning their aggression into convincing threat displays without provoking an actual physical conflict [44,45]. Moreover, convincing threat displays can pave the way to establishing social dominance [46,47]. This suggests that aggressiveness should be visible in conspicuous nonverbal cues and that displays of aggressiveness should resemble displays of dominance. Our results were in line with these expectations. Animations modified to represent dominance resembled animations representing aggressiveness. Participants also chose expansive vertical and horizontal movements when asked to create aggressive stimuli. However, in comparison to dominance, velocity played a more important role for stimuli representing aggressiveness. In short, high aggressiveness looked like high dominance but with faster movements.

People not only perceive dominance and aggressiveness but also likability, competence and trustworthiness in nonverbal cues such as body movements and facial photographs [23,30,31]. Moreover, social cognition is claimed to have its roots in two universal categories namely warmth (including trustworthiness and agreeableness) and competence [42]. We assumed that such findings and considerations also transfer to body motion and that basic social qualities are reflected in simple motion cues. In accordance with previous work, that also was based on stick-figure animations of speakers, results for competence were the least convincing [23]. It appears that competence is difficult to express through body motion or through the simple cues that were manipulated during our experiments.

In comparison to competence more impressive results were obtained for friendliness and trustworthiness. In both cases, velocity was revealed as the most influential factor. Participants associated fast movements with low trustworthiness and low friendliness. However, there was a noteworthy difference between dot- and stick-figure animations. When creating “unfriendly” stick-figure animations, participants not only made movements faster but they also made the vertical movements larger. In other words, unfriendly stick-figure animations were equipped with fast, expansive up and down movements while friendly stick-figures were equipped with slow and dampened vertical movements. This is in accordance with previous studies showing that amplitude and velocity of vertical body movements (in particular arm movements) play a prominent role in perceptions of friendliness or agreeableness [26,48,49]. There were also differences between dot-animations and stick-figure animations with regard to trustworthiness. To a certain extent dot animations of high trustworthiness were associated with low horizontal and vertical expansiveness, whereas for stick-figure animations vertical expansiveness was of minor importance. Such differences indicate that for some social categories information of body form (e.g., seeing an arm and not only a single dot moving up and down) is of greater importance than for other social categories (see below also). To examine this in more depth additional experiments with different abstractions of body form (e.g., full body versus arm only versus dot only) are required.

Some characteristics of body motion such as the flow, the velocity and other kinematic features can alter the communicative value of a gesture [11,44,49]. Our findings support this and demonstrate that people ascribe meaning to simple motion cues embedded in the behavioural stream. Similarities in the results obtained for dot- and stick-figure animations suggest that variations in motion amplitude, in velocity and in motion direction can be prominent social cues. They even have communicative value when it is not apparent that movements stem from a moving body. To classify some social qualities—in particular those that require quick behavioural responses—motion cues such as expansiveness might be more important than the specific gestures with which this expansiveness is produced. However, it should be noted that animations of single dots were based on the movements of one hand. Speakers may often use their hands to express themselves and their views. Hence, the hands may create the main portion of variation leading to similar patterns for dot- and stick-figure animations. Future work should elaborate on this and test whether the findings of the current study can be reproduced with artificially created movements [2].

It is possible that our findings result, in part, from the limited set of options participants were offered to manipulate the behaviour of the stimuli. It may have guided the participants into choosing one of these options, simply because they felt inclined to differentiate between positive and negative versions of the items we used (e.g., trustworthy versus not trustworthy). Although the random presentation order of the items and the random composition of stimuli for each session might have mitigated such effects, it requires additional experiments to rule them out. Such experiments need to extend the repertoire of motion cues thereby increasing the complexity of stimuli.

Raising complexity of the stimuli and the experimental set-up needs to be divided into several steps, because presenting too many manipulators (here: slider bars) simultaneously would make it too difficult for the participants to make clear decisions. A solution could be to conduct series of experiments with subsequent experiments building on the findings of the previous ones. Multiple studies might also include standard rating experiments, which make use of the stimuli created by a “reverse” rating. Such a series of experiments would be then divided into stimuli creation and stimuli testing phases.

The “reverse” procedure enables variations in stimulus appearance according to a fixed range of manipulations (i.e., slider bar values in this study), thereby allowing more control because changes in motion can be directly measured by the steps of the fixed range. In principle, our experimental design is in line with this. However, there was variation in the amplitude height of the source material. More specifically, the maximum amplitude for a stick-figure animation (or dot animation) was not the same for all rounds of an experimental session. The animations were composed of different raw materials and for this reason a slider bar value of seven, for instance, did not change the amplitude in the same way for all stimuli. On the one hand, the process of stimulus composition we applied was intended to create variation, in order to avoid having the same stimulus for all conditions and all participants (see Method/ Manipulation of Horizontal and Vertical Amplitudes). On the other hand, it added variation that in part undermined the rigidness of the well-defined steps of manipulations possible. All in all, the procedure we used was a compromise between controlled variability (i.e., slider bar range) and variability arising from the stimulus composition process. However, the results obtained indicate that the variance created by the manipulations exceeds the variance created by the stimulus composition procedure.

Despite the above mentioned limitations, we see several advantages in our reversed rating procedure. For instance, in a “classical” rating experiment many different stimuli are required: separate stimuli for each aspect under investigation, stimuli with different gradations of these aspects as well as different combinations of these aspects. The reverse rating procedure transforms the process of stimulus creation by transferring elemental tasks from the experimenter to the participants. People are not confronted with ready-made stimuli created in an extensive preparation phase, but they become part of the stimuli creation process itself. They are enabled to manipulate stimuli and combine different manipulations in one single step. The reverse approach thereby reduces the sample size needed to investigate the relative influence of different nonverbal cues and how they combine to form a common cue. This is particularly useful for exploratory studies with no clear hypotheses.

At first glance, the task of the reverse rating procedure appears to be technically the same as in a “classical” approach (i.e., a participant moves slider bars on a computer screen). However, enabling participants to modify a stimulus gives them immediate feedback as the effects of the manipulations and the different nuances of these manipulations are directly observable. In “classical” rating experiments, on the other hand, participants are simply observers working through a list of verbal items without receiving any kind of feedback on their actions. For this reason, participants might experience greater difficulty in perceiving these items as a coherent set of attributes that define a stimulus. Moreover, verbal items are often interrelated and can be reduced to a few categories, thereby missing important subtleties. In the reverse rating procedure, due to direct feedback, participants are better able to grasp such subtleties and the value of each component they manipulate, something the “classical” setting fails to convey. Ignoring one motion component in our set-up would massively affect the appearance of the created stimulus, while omitting an item in the “classical” rating procedure may not even be noticed by the participants. On a side note, even a small increase in the number of manipulations (i.e., additional slider bars changing additional aspects) increases the complexity of the stimuli exponentially (but see above) and this happens without the need to increase the number of participants to the same extent.

Actively changing the appearance of a stimulus by using a set of modifiers also allows a greater fine-tuning in filtering specific cues. The effect of single variables but also the way in which different variables interact and form “meaningful” patterns can be tested explicitly. Of course, the “reverse” procedure does not need to be confined to motion cues. A great deal of nonverbal information from different sources (e.g., facial cues, vocal cues, etc.) that play a role in social perception can be investigated this way. For instance, it should be possible to manipulate the features of voices in similar ways as was done here with body motion.

At the current state of the research the insights we gained cannot be applied to all types of motion behaviours, because the stimuli were obtained from a specific source, namely political speeches. Even though previous studies that were based on stick-figure animations also obtained similar results, future work needs to make use of different sources of motion and include people from different cultural backgrounds. It has been demonstrated, for instance, that happiness—a social category that assumedly is linked to friendliness—is conveyed by higher speed of motion than sadness [50,51]. We find that high friendliness is associated with lower speed of motion than low friendliness. Although the categories friendliness and happiness are only interchangeable to a certain extent, the results appear inconsistent. As already mentioned the type of motion as well as the context in which it is shown might play a role here. It makes a difference if people are asked to categorize movements that are captured from human gait, from dancers or from public speeches. However, the “reverse” approach that we applied here might be a useful tool to uncover the commonalities and the differences between movements performed in a variety of contexts.

## Conclusions

People often extract social information from motion or motion cues. We turned the common procedure of performing rating experiments upside-down and asked people to match the movements of stick-figure animations and single dot animations to different qualities of social relevance. We found that variations in amplitude and velocity of motion can be strong communicators of social information for both types of stimuli. This indicates that some motion cues do not have to be linked to specific body parts to allow categorizations on basic social dimensions. Our experimental approach of asking people to make their own stimuli further reveals that there is a necessity to investigate the interplay of different motion cues, as different combinations of cues can alter the message conveyed.

## Supporting information

S1 Dataset R code and corresponding data to perform all statistical analyses presented in the manuscript (requires R; download R software from http://cran.r-project.org. (ZIP)

S2 Dataset Demo-version of the program used in the “reverse” rating experiments. Program runs under windows (read me file with instructions is contained in the zip-file). Dataset also contains sample of the core procedures used for stimulus creation and information on the single steps of stimulus preparation.
(ZIP)

S1 Video Stick-figure reference position as well as stick-figures with high vertical and high horizontal expansiveness. Blue stick-figure belongs to a subset of stick-figures showing only expansive vertical movements. Orange stick-figure belongs to a subset of stick-figures showing only expansive horizontal movements. Grey-stick-figure provides the reference points for interpolation (i.e., weighted mean between coordinates of blue or orange stick-figure and coordinates of the grey stick-figure).
(MP4)

S2 Video Stick-figure mean position on the basis of stick-figures with high vertical and high horizontal expansiveness. Blue stick-figure belongs to a subset of stick-figures showing only expansive vertical movements. Orange stick-figure belongs to a subset of stick-figures showing only expansive horizontal movements. Black-stick-figure represents the mean between the x-coordinates of the standard-stick figure (see S2 Video and Fig 2) and the orange stick-figure as well as the mean between the y-coordinates of standard stick-figure and the blue stick-figure.
(MP4)
